# Diagnostic performance of adult-based ultrasound risk stratification systems in pediatric thyroid nodules: a systematic review and meta-analysis

**DOI:** 10.3389/fendo.2023.1187935

**Published:** 2023-05-12

**Authors:** Zhichao Xing, Yuxuan Qiu, Jingqiang Zhu, Anping Su, Wenshuang Wu

**Affiliations:** ^1^ Center of Thyroid and Parathyroid Surgery, West China Hospital, Sichuan University, Chengdu, China; ^2^ Laboratory of Thyroid and Parathyroid Disease, Frontiers Science Center for Disease-related Molecular Network, West China Hospital, Sichuan University, Chengdu, China; ^3^ Department of Ultrasound, West China Hospital, Sichuan University, Chengdu, China

**Keywords:** pediatric thyroid nodules, risk stratification systems, ultrasonography, diagnostic performance, meta-analysis

## Abstract

**Purpose:**

Ultrasound (US) is the first choice in the detection of thyroid nodules in pediatric and adult patients. The purpose of this study was to evaluate the diagnostic performance of adult-based US risk stratification systems (RSSs) when applied to the pediatric population.

**Methods:**

Medline, Embase, and Cochrane Library (CENTRAL) were searched up to 5 March 2023 for studies about the diagnostic performance of adult-based US RSS in pediatric patients. The pooled sensitivity, specificity, positive likelihood ratio (LR), negative LR, and diagnostic odds ratio (DOR) were calculated. The summary receiver operating characteristic (SROC) curves and area under the curve (AUC) were also analyzed.

**Results:**

The sensitivity was highest in American College of Radiology-Thyroid Imaging Reporting and Data System (ACR-TIRADS) category 4–5 and American Thyroid Association RSS high-intermediate risk (ATA), which was 0.84 [0.79, 0.88] and 0.84 [0.75, 0.90], respectively. The specificity was highest in ACR-TIRADS category 5 and Europe-TIRADS (EU-TIRADS) category 5, which was 0.93 [0.83, 0.97] and 0.93 [0.88, 0.98], respectively. The ACR-TIRADS, ATA, and EU-TIRADS showed moderate diagnostic performance in pediatric thyroid nodule patients. For Korea-TIRADS (K-TRADS) category 5, the summary sensitivity and specificity with a 95% CI were 0.64 [0.40, 0.83] and 0.84 [0.38, 0.99], respectively.

**Conclusions:**

In conclusion, the ACR-TIRADS, ATA, and EU-TIRADS have moderate diagnostic performance in pediatric thyroid nodule patients. The diagnostic efficacy of the K-TIRADS was not as high as expected. However, the diagnostic performance of Kwak-TIRADS was uncertain because of the small sample size and small number of studies included. More studies are needed to evaluate these adult-based RSSs in pediatric patients with thyroid nodules. RSSs specific for pediatric thyroid nodules and thyroid malignancies were necessary.

## Introduction

Thyroid cancer is the most common pediatric endocrine cancer and presents a diagnostic challenge in pediatric populations. The reported prevalence of thyroid nodules is 3.1% in adolescents (1). However, the malignancy rate is estimated to be 22–26% in children with thyroid nodules and 5–10% in adults (2–5). Furthermore, pediatric patients are more likely to present with cervical lymph node metastases (40–80%) and distant metastases (20–30%) such as pulmonary metastases than adults (6, 7). Therefore, early and accurate diagnosis in children is extremely important.

Neck ultrasound (US) is the first choice in the detection of thyroid nodules in pediatric and adult patients (8–10). Adult-based neck US risk stratification systems (RSSs) have been developed in recent years to integrate US features and improve diagnostic accuracy as an aid in the stratification of the risk of malignancy, such as the American College of Radiology–Thyroid Imaging Reporting And Data System (ACR-TIRADS), American Thyroid Association Ultrasound Risk Stratification Systems (ATA RSS), European Thyroid Imaging and Reporting Data System (EU-TIRADS), Korean Thyroid Imaging Reporting and Data System (K-TIRADS), and Kwak Thyroid Imaging Reporting and Data System (Kwak-TIRADS) (11–15).

Korean Professor Jin Young Kwak was the first in the world to propose a practical TIRADS to categorize thyroid nodules and stratifying their risk of malignancy in 2011, which we called Kwak-TIRADS now (15). He suggested that the following US features showed a significant association with malignancy: solid component, hypoechogenicity, marked hypoechogenicity, microlobulated or irregular margins, microcalcifications, and taller-than-wide shape. Risk stratification of thyroid malignancy by using the number of suspicious US features allows for a practical and convenient Kwak-TIRADS.

However, we do not have any formalized, US-based RSS in pediatrics. Recently, a few studies have reported the utility of these adult-based RSSs in pediatric patients. However, pediatric thyroid cancers are different in clinical, molecular, and pathologic characteristics from those in adults. These RSSs depend significantly on nodule size, while thyroid volume increases with age, and nodule size is not predictive of malignancy in pediatric patients. Therefore, the appropriateness of these RSSs remains to be explored when applied to pediatric patients.

Therefore, the purpose of this study was to evaluate the diagnostic performance of the adult-based RSS when applied to the pediatric population and provide information to guide future clinical practice.

## Methods

The meta-analysis was reported in accordance with the instructions of the Preferred Reporting Items for Systematic Reviews and Meta-analysis (PRISMA) extension statement incorporating network meta-analyses (16, 17).

### Search strategy

The Medline, Embase, and Cochrane Controlled Register of Trials (CENTRAL) and Web of Science databases were searched up to March 5, 2023. The search terms to retrieve related studies were as follows: [(thyroid) AND (thyroid imaging reporting and data system)] OR [(thyroid image reporting and data system) OR (TIRADS) OR (TI-RADS) OR (RSS) OR (guideline)] AND [(pediatric) OR (adolescent) OR (child) OR (children)]. Two investigators independently checked retrieved articles blinded to the journal, author, and so on. All abstracts to obtain possible applicable articles and the full text were screened to determine the final eligible articles. Relevant reviews and their reference list were also checked. Discrepancies were resolved by discussion with another investigator.

### Inclusion criteria

(a) The study was based on the diagnostic performance of adult-based ultrasound RSS, such as ACR-TIRADS, ATA, EU-TIRADS, K-TIRADS, and Kwak-TIRADS. (b) The patients were pediatric with thyroid nodules. (c) The reference standard was based on pathological diagnosis or imaging follow-up. (d) Data available for sensitivity, specificity, positive predictive value (PPV), negative predictive value (NPV), and diagnostic accuracy. (e) The language was limited to English.

### Exclusion criteria

(a) Letters, editorials, conference abstracts, and review articles. (b) The topics of articles were not about the diagnostic performance of adult-based ultrasound RSS. (c) The patients were not pediatric. (d) If studies had an overlapping population, we included the study with the largest population and excluded others.

### Data extraction

The eligible articles were reviewed, and the relevant data were extracted using a standardized form. (a) Study characteristics: first author, year of publication, country or region, study period, study design, sample size, and reference standard; (b) Patient characteristics: number of patients, mean age, and male-to-female ratio; (c) Diagnostic performance: numbers of total thyroid nodules, numbers of true positive (TP), true negative (TN), false positive (FP), false negative (FN) thyroid nodules, sensitivity, specificity, PPV, NPV, and diagnostic accuracy; (d) Standard reference: biopsy pathology, surgery pathology, and follow-up; (e) US examinations: US model and vendor, number of readers, and experience.

### Quality assessment

Two reviewers assessed the quality of the included articles independently using Quality Assessment of Diagnostic Accuracy Studies-2 (QUADAS-2) (18), and disagreement was resolved by discussion. This tool is composed of four domains: patient selection, index test, reference standard, flow, and timing. Each domain is assessed according to bias. Risk of bias was judged as “low,” “high,” or “unclear.” The first three domains are assessed in terms of concerns regarding applicability.

### Statistical analysis

Statistical analysis was mainly performed using Stata version 15.0 software (StataCorp, LLC; College Station, TX). A value of *p* < 0.05 was taken to indicate statistical significance.

The pooled sensitivity, specificity, positive likelihood ratio (LR), negative LR, and diagnosis odds ratio (DOR), each has 95% confidence intervals (95% CI), were calculated using a bivariate random-effects model, and a coupled forest plot was constructed. In addition, a hierarchical summary receiver operating characteristics (HSROC) curve with 95% confidence and prediction regions was plotted and area under the curve (AUC) was also analyzed. The criteria for the positive test results were set to be (a) RSS category 5 or (b) RSS category 4 or 5. For example, if we set category 5 as a cutoff value, TP nodules indicated the nodules classified as category 5 on US and turned out to be malignant. We followed the reference standard set in each study.

Heterogeneity was assessed using the Higgins inconsistency index (*I* ^2^) test with a value > 50%, indicating the presence of heterogeneity, and a coupled forest plot was used to graphically assess the presence of a threshold effect (a positive correlation between sensitivity and false-positive rate among the selected studies). We regarded *I* ^2^ > 50% or *P*-value of *Q*-test < 0.05 as high heterogeneity. Among the potential covariates such as sample size, region, standard reference of malignant nodules, and standard reference of benign nodules, we compared “sample size more than median” vs. “sample size less than median,” “America studies” vs. “Europe studies,” “surgery and/or biopsy pathology” vs. “surgery pathology” for malignant nodules, “surgery and/or biopsy pathology and follow-up” vs. “surgery and/or biopsy pathology” for benign nodules.

## Results

### Literature search

**Figure 1:**
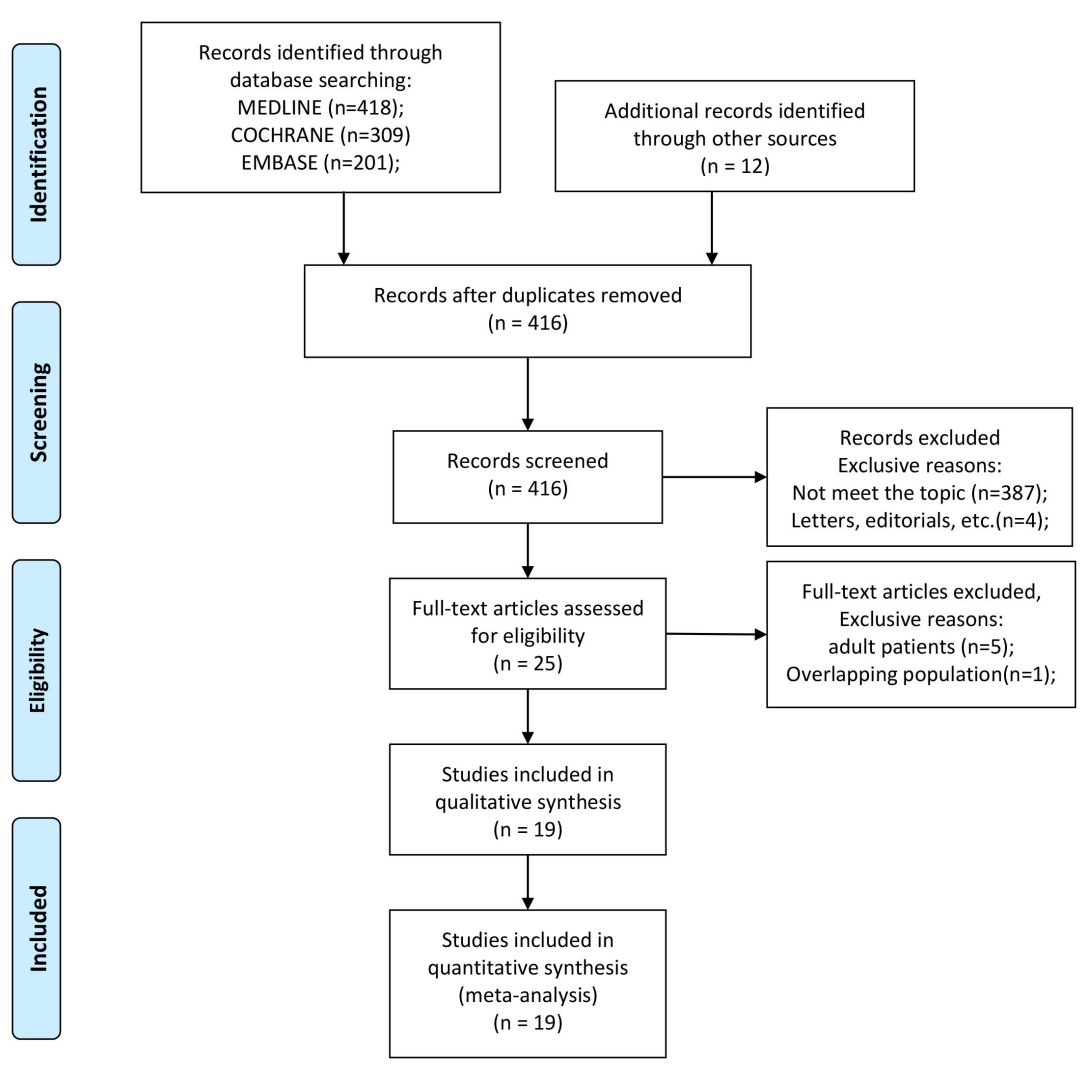
PRISMA diagram of study selection.

The details of article screening procedures were as **Figure 1**. A total of 940 articles were generated using search terms mentioned above, and 524 were removed because of duplications. We excluded 387 that did not meet the topic of our study, and four letters, editorials, conference abstracts, review articles after reviewing the titles and abstracts. The remaining 25 articles were screened for eligibility seriously, and five were abandoned because the assessment of diagnosis performence is based on adult patient and one study had an overlapping population. Finally, the remaining 19 studies were included in our meta-analysis.

### Characteristics of studies

The characteristics of included studies were detailed in **Table 1** (19–37). All the studies were retrospective. The overall study period was from 1996 to 2021. A total of 1,927 pediatric thyroid cancer patients and 2,263 modules were included. Ages ranged from 0.9 to 22 years. Male patients accounted for 23.1% and female for 76.9%. This study included 660 malignant nodules and 1,603 benign nodules. All malignant nodules and most of benign nodules in the included studies have been diagnosed by surgical pathology or biopsy. Benign nodules included only in six studies were diagnosed by biopsy pathology, surgery pathology, or at least 1 year of follow-up (19–21, 27, 31, 35).

**Table 1:** Characteristics of the included studies.

First author	Year	Study period	Country	Study design		No. of patients	M	F		Age	Range/SD	Inclusion age (years)
Lim-Dunham	2017	1996–2016	USA	R		33	5	28		16 in benign; 16.5 in malignant	<=18	
Creo	2018	1996–2015	USA	R		112	16	96		15.5	15.5 ± 3.2	<=18
Martinez-Rios	2018	Jan. 1992–Oct. 2015	Canada	R		124	40	84		13.6	3.3–17.7	<21
Zaltsberg	2019	Aug. 2007–Aug. 2017	Canada	R		75	16	59		13.4	3–18	<18
Lim-Dunham	2019	1996–2017	USA	R		62	6	56		12.5 in male; 16.5 in female	<=18	
Polat	2019	2015–2018	Turekey	R		106	35	71		11.4	1–17	<18
Uner	2019	NA	Turekey	R		64	10	58		15.2	3–18	<=18
Richman	2020	Jan. 2004–Jul. 2017	USA	R		314	54	260		14.9	14.9 ± 2.7	<=18
Arora	2020	2008–2018	USA	R		20	4	16		14.9	7–22	<=22
Scappaticcio	2021	Jan. 2017–Mar. 2021	Italy	R		36	10	26		15	11–17	<19
Piccardo	2021	Jan. 2012–Dec. 2017	Italy	R		52	20	32		17	15–18	<=18
Ahmad	2021	Jan. 2015–Mar. 2019	USA	R		115	25	90		15.5	5.0–20.2	<=21
Fernández	2021	2005–2020	Spain	R		24	6	18		15.3	13.3–17.3	<18
Lee	2021	Aug. 2007–Feb. 2020	Korea	R		107	24	83		13.9	4–18	<19
Tuli	2022	2000–2020	Italy	R		200	81	119		12	2–18	<18
Yang	2022	Jan. 2004–Sept. 2020	USA	R		139	20	119		17.5	15.3–19.3	<=21
Borysewicz-Sanczyk	2022	NA	Poland	R		17	4	13		15.3	5–18	5–18
Daniels	2022	2007–2018	USA	R		106	20	86		15.6	0.9–18.8	<19
Kim	2022	Jan. 2000–Apr. 2020	Korea	R		221	49	172		16	13–17	<=21
No. of nodules	Mal	Ben	Median/mean nodule size (cm)			RSS	reference standard			US model (vendor)	Interpretation	
			Ben		Mal		Ben		Mal		No. of readers	Experience (y)
33	12	21	2.1		2.55	ATA	s/b		s	Acuson Sequoia 512, XP128, Aspen (Siemens), Logic E9 (GE)	2	>10
145	50	95	NA		NA	ATA	s/b/f (1 year)		s/b	NA	2	sum>27
123	52	71	2.75			ATA, Kwak	s/b/f (2 years)		s/b	iU22 (Philips), Aplio (Toshiba)	3	2-37
300	52	248	NA		NA	ACR, Kwak	s/b/f (1 year)		s/b	iU22 (Philips), Aplio 500 (Toshiba)	4	5-20
33	12	21	1.9			ACR	s/b		s	Acuson Sequoia 512, XP128, Aspen (Siemens), Logic E9 (GE)	2	>10
105	5	100	0.74			ACR	s/b/f (1 year)		s/b	Aplio 500 (Toshiba), RS80A with Prestige (Samsung Medison)	2	3-7
68	19	49	0.8			ACR	s/b		s/b	iU22 (Philips), Apolio (Toshiba)	NA	NA
404	77	327	NA		NA	ACR	s/b		s/b	Acuson Sequoia (Siemens), Logiq E9 (GE)	4	6-33
20	7	13	NA		NA	ACR	s/b/f (2 years)		s/b	NA	NA	NA
41	12	29	10 (7-13)			ACR, EU, K, ATA	s/b		s	MyLabTMSix, Esaote	3	NA
52	14	38	13 (11-12)			ACR, EU, ATA	s/b		s	LOGIQ S8 (General Electric Medical Systems)	3	NA
138	10	128	NA		NA	ACR, PED, ATA	s/b		s/b	NA	2	NA
19	7	12	19 (9-36)		22.2 (15-34)	EU	s/b		s/b	NA	2	NA
133	62	71	NA		NA	K	s/b		s/b	Aplio XG (Toshiba), iU22 (Philips), Aixplorer (SuperSonic), Logiq E9 (GE)	2	6-8
200	26	174	8 (8-10)		24 (7-60)	ACR, EU	s/b		s/b	NA	3	NA
139	56	83	2.4 (1.6-3.7)			ACR	s/b		s/b	NA	3	1-30
16	5	11	2.0–22.6 (9.9 ± 6.95)		4.5–19.0 (13.1 ± 5.86)	ATA, BTA	s/b		s/b	Apolio (Toshiba)	1	NA
106	59	47	NA		NA	ACR	s/b/f (2 years)		s/b	NA	2	4-11
221	135	86	NA		NA	ACR, ATA, EU, K, AACE/ACE/AME	s/b		s/b	iU22 and EPIQ 5 (Philips), Aplio XG (Toshiba)	3	1-8

R, retrospective study; ACR, American College of Radiology TIRADS; ATA, American Thyroid Association Ultrasound Risk Stratification Systems; EU, European TIRADS; K, Korean TIRADS; Kwak, Kwak TIRADS; PED, Pediatric TIRADS; BTA, British Thyroid Association Ultrasound Risk Stratification Systems; s, surgery pathology; b, biopsy pathology; f, follow-up; NA, not available.

### Quality assessment

**Figure 2:**
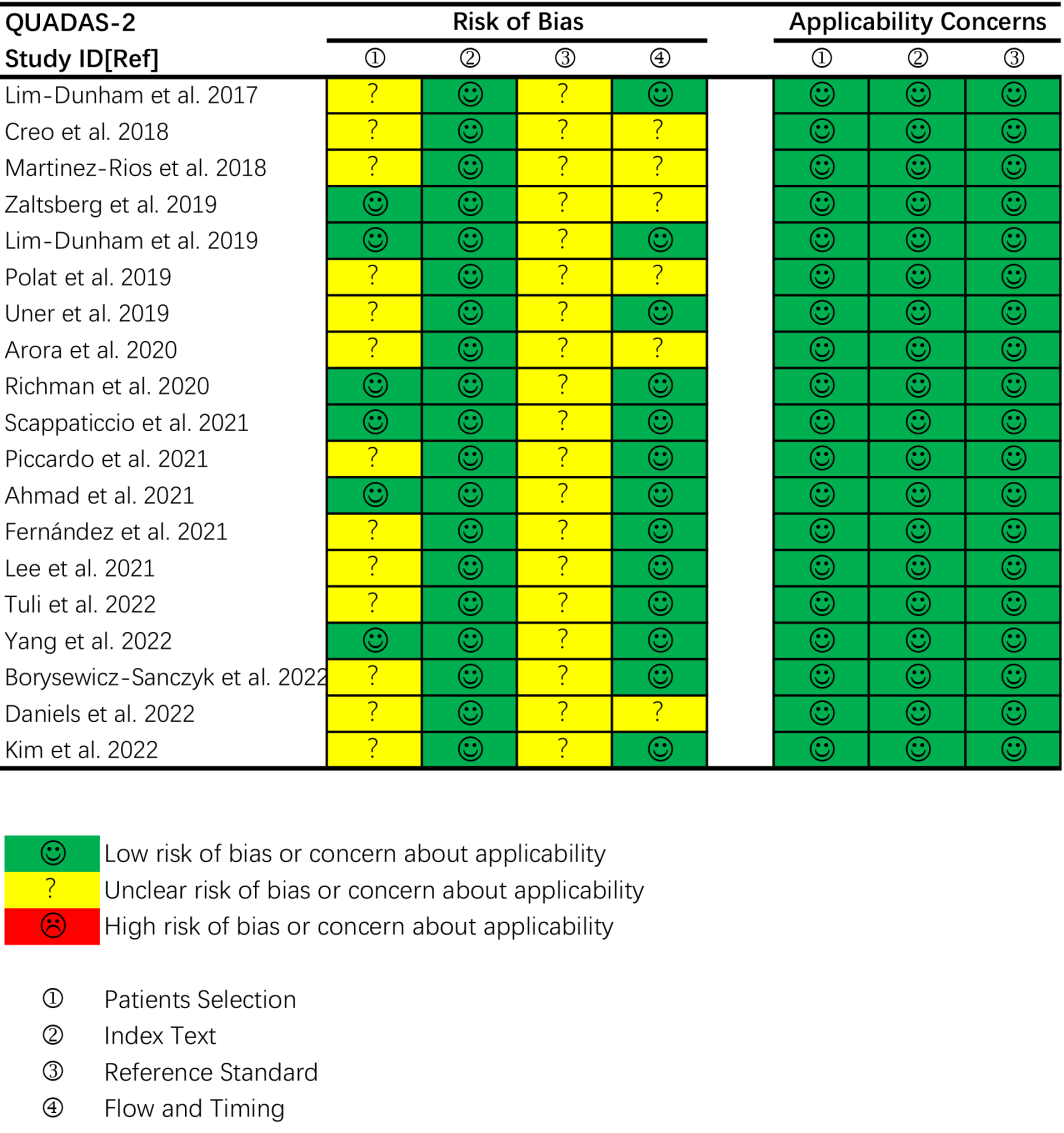
Quality assessment of the included studies according to the Quality Assessment of Diagnostic Accuracy Studies-2 (QUADAS-2) criteria.

The overall quality of the included studies assessed by QUADAS-2 was moderate. Five articles satisfied six of the seven items, and nine articles satisfied five items. The details are shown in **Figure 2**. Thirteen studies had an unclear risk of bias in patient selection. Consecutive enrollment was not clarified in 10 studies (20, 22, 24, 25, 27, 31, 32, 35–37). Martinez-Rios et al. included thyroid nodules measuring more than 10 mm (21). Tuli et al. included thyroid nodules measuring more than 5 mm (33). Piccardo et al. included patients treated with radiotherapy for nonthyroidal cancers (29). No study had an unclear risk of bias in the index test domain because of blinding to the reference standard during the US examinations. All studies had an unclear risk of bias in the reference standard domain because of no or unclear blinding to the index test during pathologic evaluation. Six studies had an unclear risk of bias in the flow and timing domain because of inconsistency or unclear consistency on the reference standard for diagnosing benign nodules across the study population (19–21, 24, 27, 35). There were no concerns regarding the applicability of the patient selection, index test and reference standard.

### Diagnostic performance

The diagnostic performance and AUC of ACR-TIRADS, ATA system, EU-TIRADS, K-TIRADS and Kwak-TIRADS was synthesized in **Table 2**.

**Table 2:** Diagnostic performance of different RSS.

RSS	No. of studies	Sensitivity	Specificity	DOR	AUC	LR+	LR-
ACR 5	13	0.57 [0.41, 0.71]	0.93 [0.83, 0.97]	17 [7, 37]	0.82 [0.79, 0.85]	7.7 [3.5, 17.0]	0.47 [0.34, 0.64]
ACR 4-5	10	0.84 [0.79, 0.88]	0.61 [0.49, 0.72]	8 [5, 14]	0.85 [0.81, 0.87]	2.2 [1.6, 2.9]	0.26 [0.20, 0.35]
ATA high	8	0.73 [0.65, 0.79]	0.73 [0.43, 0.91]	7 [2, 22]	0.76 [0.72, 0.79]	2.7 [1.1, 6.7]	0.37 [0.28, 0.49]
ATA high-intermediate	6	0.84 [0.75, 0.90]	0.55 [0.40, 0.70]	7 [4, 12]	0.82 [0.78, 0.85]	1.9 [1.4, 2.6]	0.28 [0.19, 0.42]
EU 5	3	0.45 [0.17, 0.76]	0.93 [0.88, 0.98]	17 [11, 27]	0.70 [0.33, 0.94]	10.9 [6.3, 17.9]	0.61 [0.56, 0.65]
EU 4-5	5	0.78 [0.68, 0.86]	0.48 [0.36, 0.61]	3 [2, 5]	0.71 [0.67, 0.75]	1.5 [1.2, 1.9]	0.45 [0.33, 0.62]
K 5	3	0.64 [0.40, 0.83]	0.84 [0.38, 0.99]	195 [84, 386]	0.56 [0.06, 0.95]	59.4 [16.8, 123.7]	0.29 [0.16, 0.40]
Kwak 5	2	0.10 [0.04, 0.18]	0.99 [0.97, 0.99]	43 [21, 66]	0.09 [0.05, 0.14]	3.4 [2.9, 3.9]	/
Kwak 4-5	2	0.99 [0.99, 1.00]	0.33 [0.11, 0.63]	1.6 [0.1, 3.1]	0.48 [0.03, 0.94]	/	/

RSS, risk stratification systems; ACR 5, ACR-TIRADS category 5; ACR 4-5, ACR-TIRADS category 4 or 5; ATA high, ATA high risk; ATA high-intermediate, ATA high-intermediate risk; EU 5, EU-TIRADS category 5; EU 4-5, EU-TIRADS category 4 or 5; K 5, K-TIRADS category 5; Kwak 5, Kwak-TIRADS category 5; Kwak 4-5, Kwak-TIRADS category 4 or 5; DOR, diagnostic odds ratio; AUC, area under the curve of receiver operating characteristic; LR+, positive likelihood ratio; LR-, negative likelihood ratio.

Diagnostic performance of ACR-TIRADS

**Figure 3:**
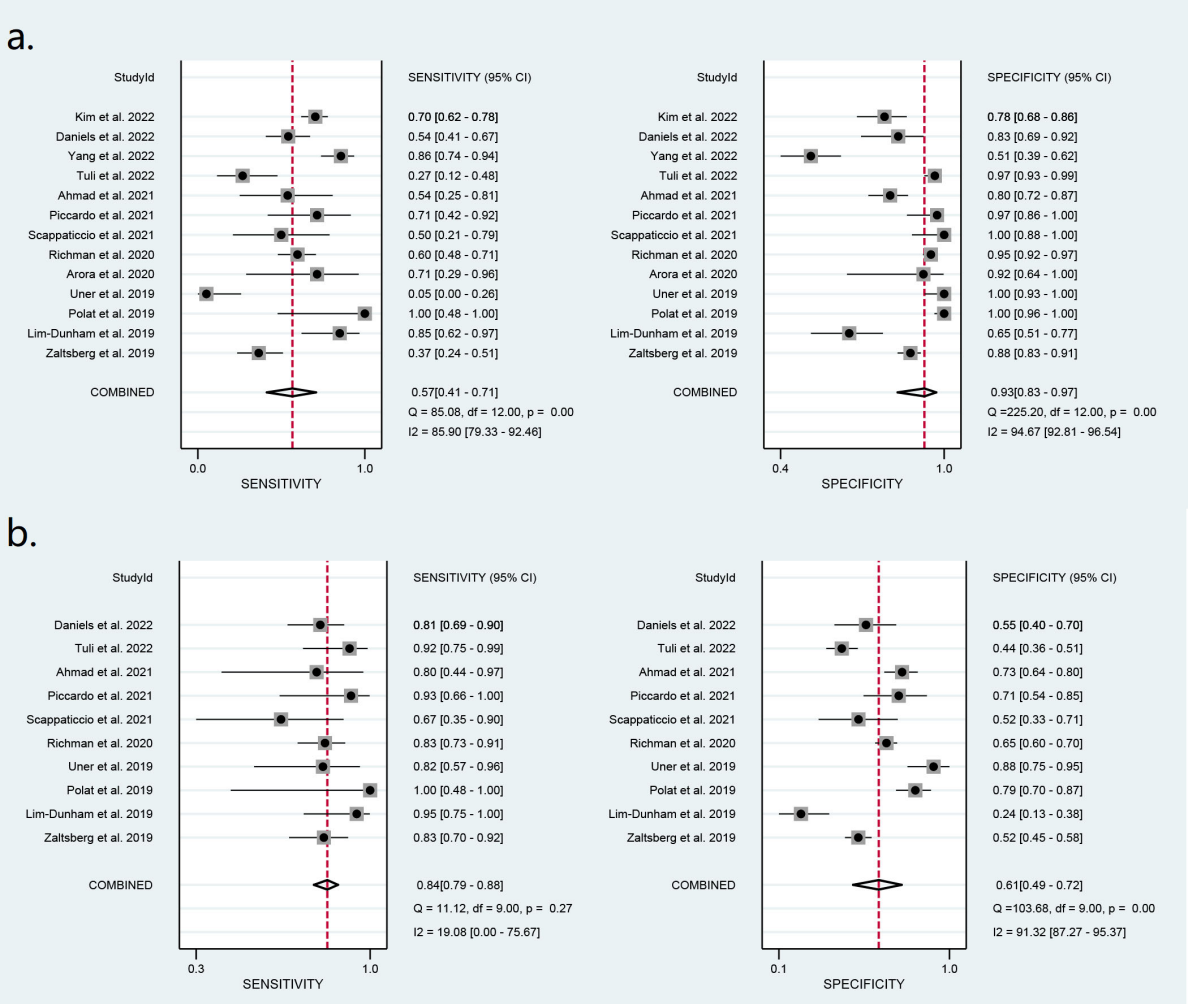
Forest plots of the pooled sensitivity and specificity for the diagnosis of malignant thyroid nodules: **(A)** ACR 5 and **(B)** ACR 4-5.

**Figure 4:**
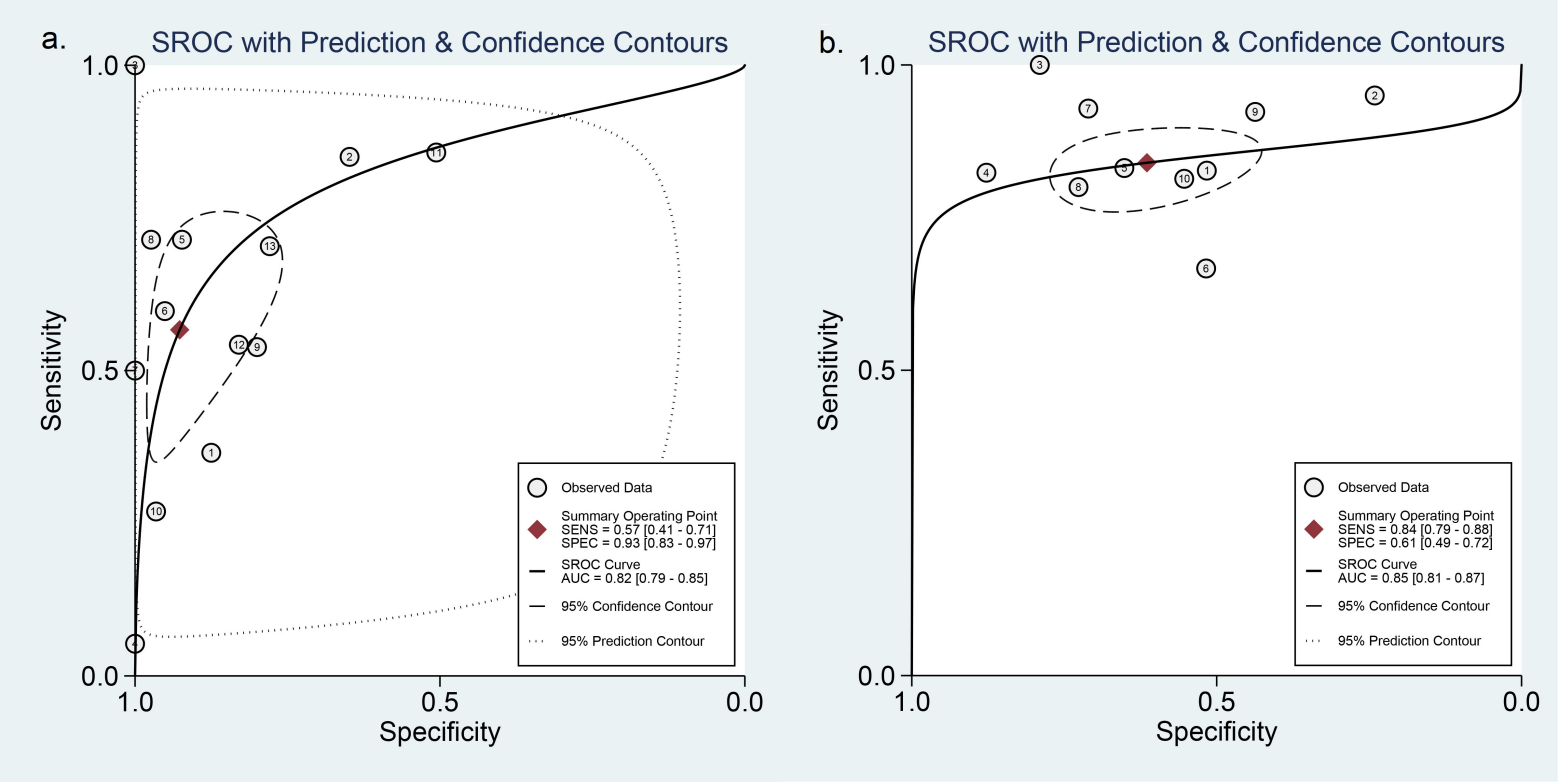
HSROC curve of the diagnostic performance: **(A)** ACR 5 and **(B)** ACR 4-5.

Thirteen studies including 1,868 nodules were pooled to analyze the diagnostic performance of ACR-TIRADS category 5 (ACR 5). As shown in **Figure 3**, the summary sensitivity and specificity with a 95% CI were 0.57 [0.41,0.71] and 0.93 [0.83, 0.97], respectively. For ACR-TIRADS category 4 or 5 (ACR 4-5), 10 studies including 1,486 nodules were pooled and analyzed, and the sensitivity and specificity were 0.84 [0.79, 0.88] and 0.61 [0.49, 0.72], respectively. The AUC was 0.82 [0.79, 0.85] for ACR 5 and 0.85 [0.81, 0.87] for ACR 4-5, shown in **Figure 4**.

### Diagnostic performance of the ATA system

**Figure 5:**
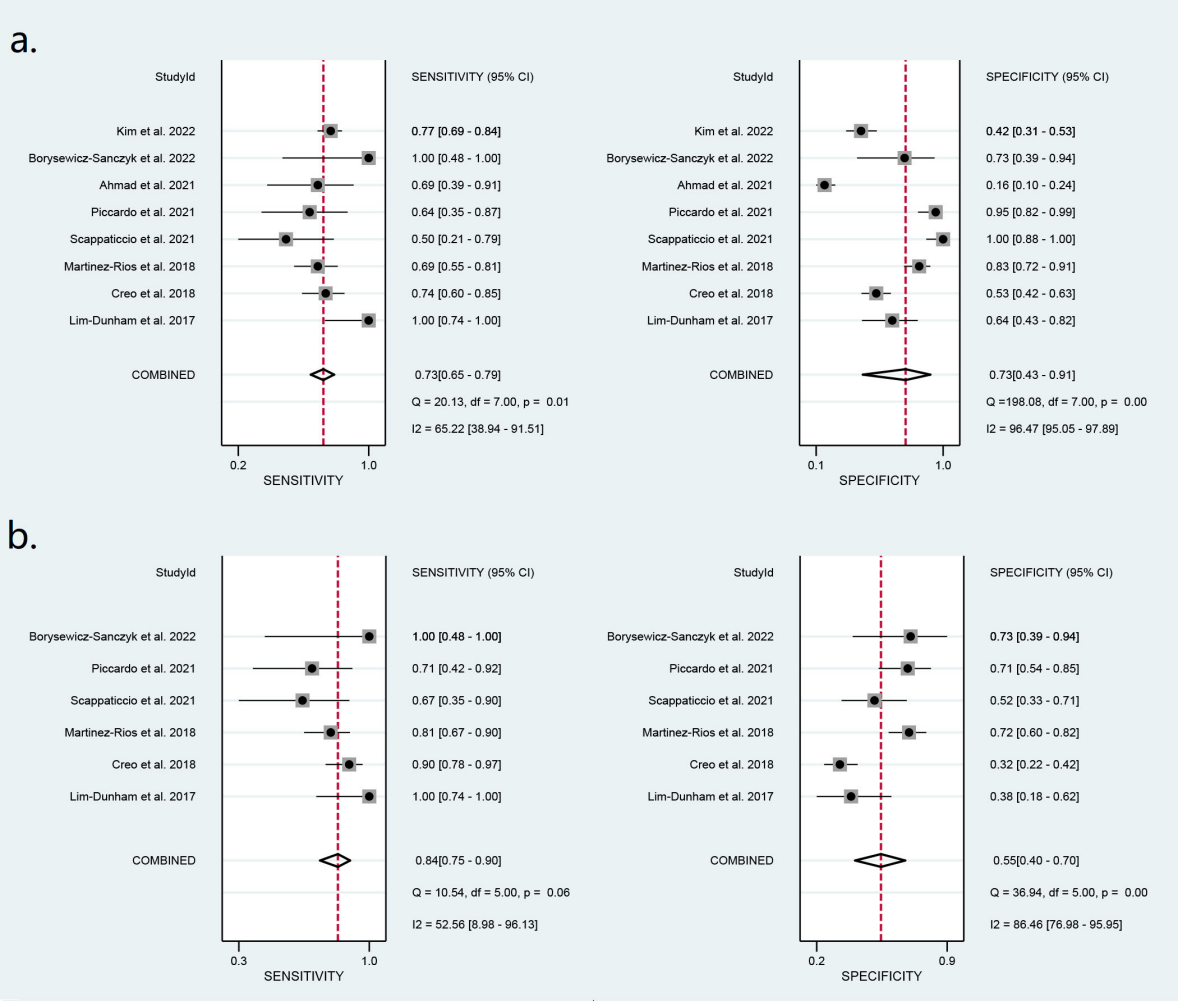
Forest plots of the pooled sensitivity and specificity for the diagnosis of malignant thyroid nodules: **(A)** ATA high risk and **(B)** ATA high-intermediate risk.

**Figure 6:**
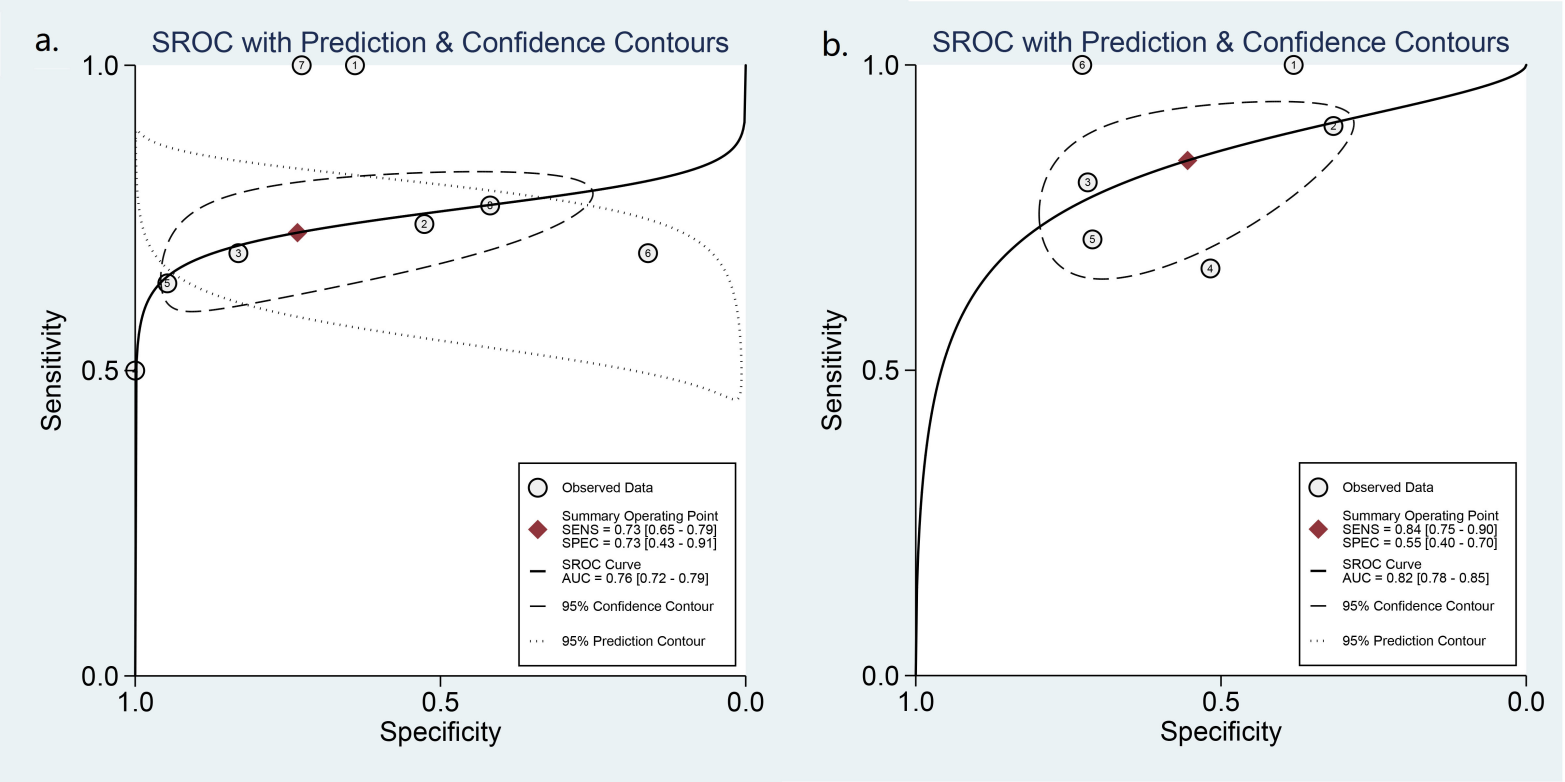
HSROC curve of the diagnostic performance: **(A)** ATA high risk and **(B)** ATA high-intermediate risk.

Eight studies including 773 ATA high-risk nodules were pooled and analyzed, and the summary sensitivity and specificity were 0.73 [0.65, 0.79] and 0.73 [0.43, 0.91], respectively. For ATA high-intermediate risk, six studies including 410 nodules were pooled and analyzed. The sensitivity and specificity were 0.84 [0.75, 0.90] and 0.55 [0.40, 0.70], respectively. The details are shown in **Figure 5**. The AUC was 0.76 [0.72, 0.79] for ATA high risk and 0.82 [0.78, 0.85] for ATA high-intermediate risk, shown in **Figure 6**.

### Diagnostic performance of EU-TIRADS

**Figure 7:**
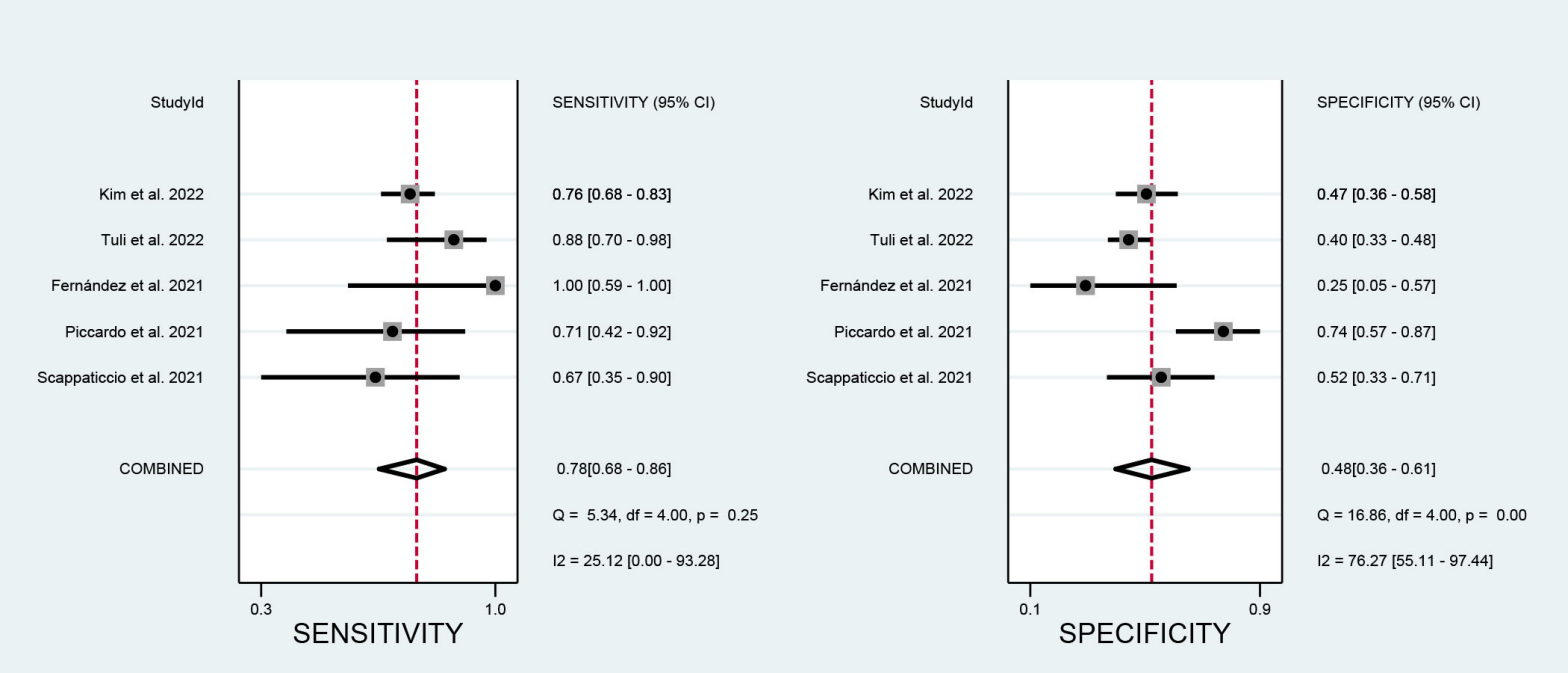
Forest plots of the pooled sensitivity and specificity for the diagnosis of malignant thyroid nodules in EU 4-5.

**Figure 8:**
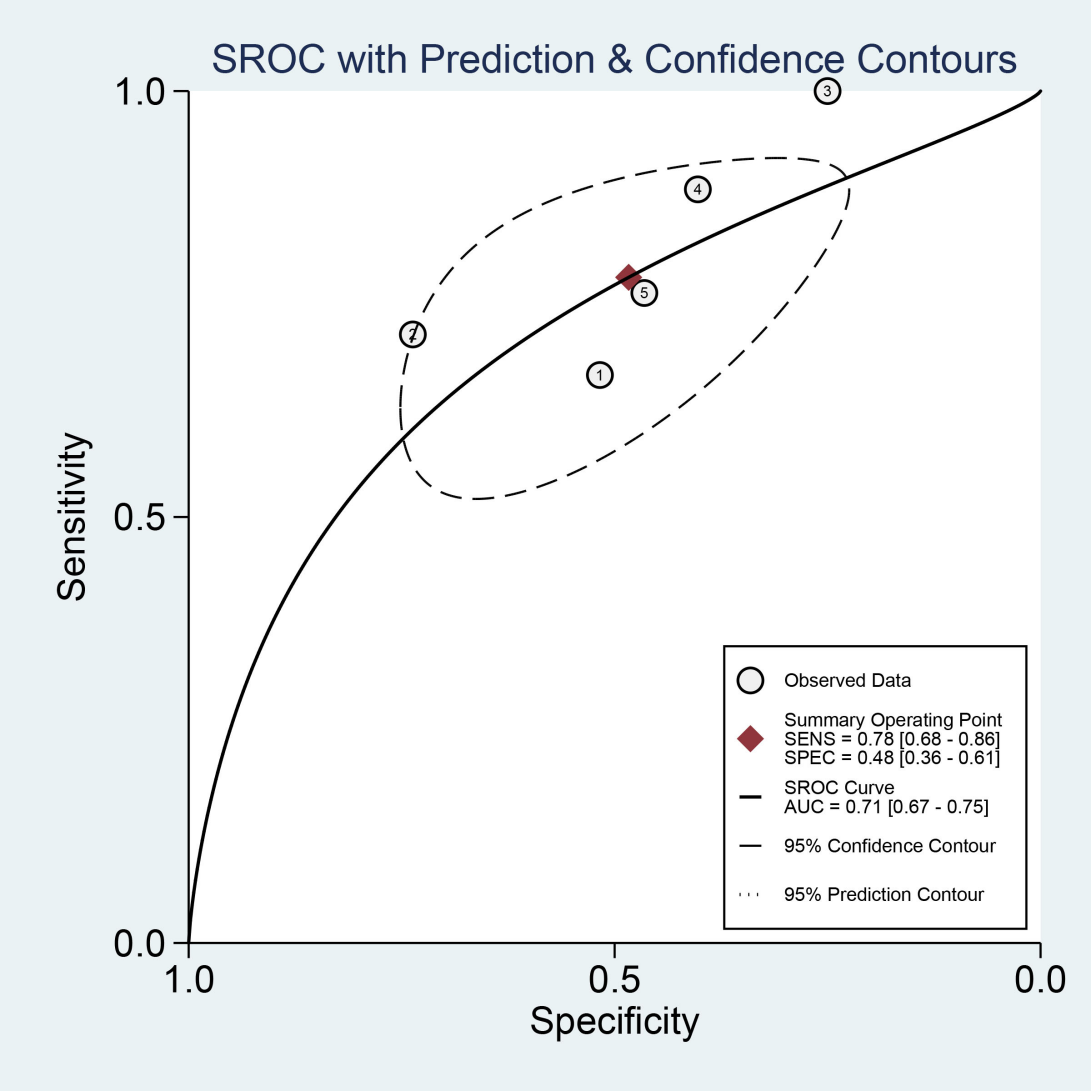
HSROC curve of the diagnostic performance of EU 4-5.

Three studies including 293 nodules of EU-TIRADS category 5 (EU 5) were pooled and analyzed. The summary sensitivity was 0.45 [0.17, 0.76], and the specificity was 0.93 [0.88, 0.98]. For EU-TIRADS category 4 or 5 (EU 4-5) shown in **Figure 7**, five studies including 533 nodules were pooled and analyzed. The sensitivity and specificity were 0.78 [0.68, 0.86] and 0.48 [0.36, 0.61], respectively. The AUC of EU TR5 was 0.70 [0.33, 0.94], and the AUC of EU 4-5 (**Figure 8**) was 0.71 [0.67, 0.75].

### Diagnostic performance of K-TIRADS

Only three studies including 385 nodules were pooled to analyze the diagnostic performance of the K-TIRADS category (K 5). The summary sensitivity and specificity with a 95% CI were 0.64 [0.40, 0.83] and 0.84 [0.38, 0.99], respectively. The AUC was 0.56 [0.06, 0.95].

### Diagnostic performance of Kwak-TIRADS

Only two studies including 423 nodules were pooled to analyze the diagnostic performance of the Kwak-TIRADS. For Kwak 5, the pooled sensitivity and specificity were 0.10 [0.04, 0.18] and 0.99 [0.97, 0.99], respectively. For Kwak 4-5, the sensitivity and specificity were 0.99 [0.99, 1.00] and 0.33 [0.11, 0.63], respectively. The AUC was 0.09 [0.05, 0.14] for Kwak 5 and 0.48 [0.03, 0.94] for Kwak 4-5.

### Meta-regression analysis

The details are shown in **Table 3**. Sample size and region might be the heterogeneous sources of specificity of the ACR 5 category. The region and standard reference for benign nodules might be the heterogeneous sources of specificity of the ACR 4-5 category. Sample size and standard reference for malignant nodules could lead to the heterogeneous specificity of the ATA high-risk category. Region resulted in the heterogeneous sensitivity of the ATA high-intermediate risk category. No potential heterogeneous source was found in the EU 4-5 category.

**Table 3:** Meta-regression analysis.

RSS	Parameter	Category	N	Sensitivity	P	Specificity	P
ACR 5	Total nodes	yes	6	0.60 [0.36, 0.85]	0.96	0.98 [0.95, 1.00]	0.03**∗**
		no	7	0.58 [0.38, 0.78]		0.86 [0.72, 1.00]	
	Region	yes	5	0.43 [0.18, 0.68]	0.21	0.99 [0.97, 1.00]	0.01**∗**
		no	7	0.65 [0.48, 0.83]		0.82 [0.72, 0.92]	
	Standard reference for benign thyroid nodules	yes	9	0.57 [0.40, 0.75]	0.99	0.92 [0.84, 1.00]	0.95
		no	4	0.55 [0.25, 0.85]		0.93 [0.83, 1.00]	
	Standard reference for malignant thyroid nodules	yes	3	0.72 [0.46, 0.98]	0.41	0.95 [0.84, 1.00]	0.41
		no	10	0.52 [0.36, 0.68]		0.92 [0.85, 1.00]	
ACR 4-5	Region	yes	5	0.86 [0.78, 0.94]	0.03**∗**	0.69 [0.54, 0.83]	0.67
		no	5	0.84 [0.78, 0.89]		0.54 [0.38, 0.70]	
	Standard reference for benign thyroid nodules	yes	7	0.85 [0.80, 0.91]	0.02**∗**	0.61 [0.46, 0.75]	0.59
		no	3	0.82 [0.73, 0.90]		0.63 [0.42, 0.84]	
	Standard reference for malignant thyroid nodules	yes	3	0.87 [0.77, 0.97]	0.15	0.48 [0.26, 0.70]	0.17
		no	7	0.83 [0.78, 0.88]		0.66 [0.54, 0.78]	
ATA high	Total nodes	yes	4	0.77 [0.62, 0.92]	0.31	0.91 [0.78, 1.00]	0.02**∗**
		no	4	0.76 [0.66, 0.85]		0.48 [0.14, 0.82]	
	Standard reference for malignant thyroid nodules	yes	3	0.72 [0.55, 0.89]	0.17	0.94 [0.83, 1.00]	0.01**∗**
		no	5	0.75 [0.67, 0.83]		0.52 [0.22, 0.82]	
ATA high-intermediate	Total nodes	yes	3	0.86 [0.72, 1.00]	0.56	0.52 [0.29, 0.75]	0.64
		no	3	0.83 [0.74, 0.93]		0.58 [0.39, 0.78]	
	Region	yes	3	0.74 [0.58, 0.91]	0.01**∗**	0.64 [0.45, 0.83]	0.44
		no	3	0.88 [0.81, 0.95]		0.47 [0.29, 0.66]	
	Standard reference for benign thyroid nodules	yes	4	0.82 [0.69, 0.95]	0.11	0.58 [0.39, 0.76]	0.84
		no	2	0.86 [0.77, 0.95]		0.52 [0.28, 0.75]	
	Standard reference for malignant thyroid nodules	yes	3	0.80 [0.65, 0.94]	0.05	0.54 [0.33, 0.76]	0.83
		no	3	0.86 [0.77, 0.95]		0.56 [0.35, 0.77]	
EU 4-5	Total nodes	yes	2	0.81 [0.61, 1.00]	0.86	0.40 [0.16, 0.63]	0.57
		no	3	0.76 [0.66, 0.87]		0.52 [0.36, 0.68]	
	Standard reference for malignant thyroid nodules	yes	2	0.69 [0.51, 0.88]	0.11	0.64 [0.52, 0.76]	0.08
		no	3	0.80 [0.68, 0.92]		0.42 [0.35, 0.48]	

RSS, risk stratification systems, ∗p < 0.05

Total nodes “yes,” sample size more than median; total nodes “no,” sample size less than median;

Region “yes,” American studies; Region “no,” European studies;

Standard reference of malignant thyroid nodules “yes,” surgery and/or biopsy pathology; standard reference of malignant thyroid nodules “no,” surgery pathology;

Standard reference of benign thyroid nodules “yes,” surgery and/or biopsy pathology and follow-up; standard reference of benign thyroid nodules “no,” surgery and/or biopsy pathology.

## Discussion

The goal of this study was to investigate the reliability and diagnostic performance of the adult-based TI-RADS in the pediatric population. We analyzed the diagnostic performance of the ACR-TIRADS, ATA RSS, EU-TIRADS, K-TIRADS, and Kwak-TIRADS in this study. Since the included studies were not paired studies, we could not directly compare diagnostic performances between different RSSs and calculate p values, which would not be statistically justified.

The sensitivity was highest in ACR category 4–5 and ATA high-intermediate risk, which was 0.84 [0.79, 0.88] and 0.84 [0.75, 0.90], respectively. The specificity was highest in ACR category 5 and EU category 5, which was 0.93 [0.83, 0.97] and 0.93 [0.88, 0.98], respectively. ACR-TIRADS, ATA RSS and EU-TIRADS showed moderate diagnostic performance in pediatric thyroid nodules with category 4-5 AUCs of 0.85 [0.81, 0.87], 0.82 [0.78, 0.85], and 0.71 [0.67, 0.75], respectively.

Although ACR-TIRADS, ATA RSS, and EU-TIRADS have moderate diagnostic performance in pediatric thyroid nodule patients. They also have some limitations.

The ACR-TIRADS subdivides features and adds points for composition, echogenicity, shape, margins, and echogenic foci, and stratifies TIRADS level based on the total points of the 5 categories of ultrasound features. This requires a high level of experience and skill, which may be difficult for primary care physicians to master and perform (38).

ATA RSS assesses the malignancy of thyroid nodules based on the performance of ultrasound features with high diagnostic weight, which improves the detection rate of malignant nodules, but has the disadvantage that the assessment of the risk of nodule malignancy is overly dependent on the stratification of suspicious ultrasound features. A small number of pediatric patients cannot be categorized according to ATA RSS because of their specific imaging presentation, and such poorly classified nodes could lead to misdiagnosis or underdiagnosis of malignant nodes (39).

ACR-TIRADS, ATA considers FNAB only for nodules greater than or equal to 10 mm, which may miss some malignant nodes in pediatric patients because thyroid volume increases with age, and nodule size is not predictive of malignancy in pediatric patients.

The EU-TIRADS concept of malignancy stratification of thyroid nodules has some similarities with the ATA guidelines. Comparatively, EU-TIRADS has a more streamlined classification of diagnostic weights for malignant nodule features, focusing on the diagnostic weights of highly specific suspicious malignant features, and has a better specificity in identifying benign and malignant nodules. However, the classification of low- and intermediate-risk nodules (EU-TIRADS 3 and 4) by EU-TIRADS explicitly requires the ultrasound features of ovoid shape and smooth margins, while some pediatric patients in the clinic do not have the above two ultrasound features meanwhile cannot be clearly classified in EU-TIRADS 5 categories. This may result in unclassifiable or subjective empirical misjudgment of risk level and is an important reason for the low sensitivity (40).The diagnostic performance of the K-TIRADS was not as expected, with an AUC of only 0.56 [0.06, 0.95]. The K-TIRADS was first proposed by the Korean Society of Thyroid Radiology and Korean Thyroid Association in 2016. Although it shows respectable diagnostic performance for thyroid nodules in adults, recent adult-based studies revealed that in comparison with ACR-TIRADS, the 2016 K-TIRADS demonstrated higher sensitivity (94.5 [92.4, 96.6] vs. 74.7 [70.7, 78.7]) but lower specificity (26.4 [24.2, 28.6] vs. 67.3 [65.0, 69.7]) (41). In this context, the modified K-TIRADS was published in 2021 (42). For pediatric populations, 2021 K-TIRADS newly recommends biopsy of nodules of 0.5–1.0 cm with high suspicion. Compared with the 2016 K-TIRADS, the 2021 K-TIRADS (biopsy cutoffs, 0.5 cm for K-TIRADS 5; 1.0–1.5 cm for K-TIRADS 4) showed higher sensitivity (34.0% vs. 67.3%; *p* < 0.001) while maintaining specificity (89.4% vs. 88.2%; *p* = 0.790) in small nodules of pediatric patients and higher specificity (5.9% vs. 25.4%; *p* < 0.001) while maintaining sensitivity (100% vs. 98.7%; *p* = 0.132) in large nodules of pediatric patients (43).

In addition, two articles investigated the diagnostic performance of the Kwak-TIRADS (19, 21). Shapira et al. reported an AUC of 0.74 [0.67–0.82] for the diagnostic performance of Kwak-TIRADS compared with 0.72 [0.61–0.82] for ACR-TIRADS. No significant difference was obtained when comparing the Kwak-TIRADS to the ACR TI-RADS (19). Martinez-Rios et al. evaluated the performance of the Kwak-TIRADS and the ATA RSS in assessing thyroid nodules in children. They showed that the test characteristics of both methods were similar to those in adults (21). However, probably because only two studies on Kwak-TIRADS were included, the results of diagnostic performance that we pooled for analysis in our study were not very meaningful.

Additionally, Borysewicz-Sanczyk et al. evaluated the ATA RSS and British Thyroid Association (BTA) ultrasound RSS in the management of thyroid nodules in pediatric patients. The sensitivity and specificity of ATA high risk were (5/5) 100% and (8/11) 72.7%, respectively, while they were (4/5) 80% and (9/11) 81.8% for BTA category 5 (37). Both RSSs showed good diagnostic performance.

We acknowledge that there were certain limitations. First, all the included studies were retrospective. Second, the number of studies on K-TIRADS and Kwak-TIRADS was small, which resulted in the pooled analyzed diagnostic performance not being very informative.

In conclusion, the ACR-TIRADS, ATA, and EU-TIRADS have moderate diagnostic performance in pediatric thyroid nodule patients. The diagnostic efficacy of the K-TIRADS was not as high as expected. However, the diagnostic performance of Kwak-TIRADS was uncertain because of the small sample size and small number of studies included. More studies are needed to evaluate these adult-based RSSs in pediatric patients with thyroid nodules. RSS specific for pediatric thyroid nodules and thyroid malignancies were necessary.

## Data availability statement

The original contributions presented in the study are included in the article/supplementary material. Further inquiries can be directed to the corresponding authors.

## Author contributions

ZX conceived the meta-analysis. All authors contributed to the development of the selection criteria, the risk of bias assessment strategy, and data extraction criteria. ZX and YQ developed the search strategy, performed database search, acquired the data, analyzed the data, and drafted the manuscript. AS and WW did statistical analysis. All authors contributed to the article and approved the submitted version.
